# Contribution of the intracellular C terminal domain to regulation of human P2X1 receptors for ATP by phorbol ester and Gq coupled mGlu_1α_ receptors

**DOI:** 10.1016/j.ejphar.2010.11.039

**Published:** 2011-03-05

**Authors:** Hairuo Wen, Richard J. Evans

**Affiliations:** Department of Cell Physiology & Pharmacology, University of Leicester, Leicester, LE1 9HN, UK

**Keywords:** P2X receptor, Ion channel, Phorbol ester, Regulation, Mutagenesis, ATP

## Abstract

P2X1 receptors are expressed in arteries and blood platelets, play an important role in the cardiovascular system, and their activity can be potentiated following stimulation of Gq coupled receptors or phorbol ester treatment. The contribution of the intracellular carboxy terminus of the P2X1 receptor to this regulation was determined using over-expression of the C terminus and a mutagenesis based approach on recombinant receptors expressed in *Xenopus* oocytes. PMA induced potentiation of P2X1 receptor currents (~ 125% above control) was abolished following over-expression of the intracellular carboxy terminus of the P2X1 receptor. To determine the molecular basis of regulation by the carboxy terminus a series of individual cysteine point mutations between His^355^ and Tyr^370^ was characterized. PMA potentiation was abolished for the P2X1 receptor mutants H355C, P358C, Y363C, K367C, F368C, K369C and Y370C. When these mutations were introduced into the carboxy terminus fragment the inhibitory effect was absent only for P358C, K367C and Y370C mutants. These results suggest that residues Pro^358^, Lys^367^ and Tyr^370^ are involved in the sequestering effect of the carboxy terminal fragment and indicate they are directly involved in modulation of the receptor by binding to a regulatory factor. The other mutants that abolished the PMA effect when introduced into the P2X1 receptor are likely to be involved in transduction of the regulatory event. These studies highlight the importance of the carboxy terminus in determining the properties and regulation of the P2X1 receptor and suggest that the intracellular terminal regions of the receptor close to the transmembrane segments interact.

## 1 Introduction

ATP gated P2X receptors comprise a distinct family of ion channels with two transmembrane segments, intracellular amino and carboxy termini and a large extracellular ligand binding loop (Young, 2009). Seven human P2X receptor subunits have been identified (P2X1–7) and these form functional homo- and hetero-trimeric receptors with a range of properties (for review see (North, 2002)). The P2X1 receptor is expressed in smooth muscle and contributes to neuronal control of the vas deferens (Mulryan et al., 2000), arteries (Vial and Evans, 2002) and the bladder (Vial and Evans, 2000). In addition, the P2X1 receptor is expressed by platelets (MacKenzie et al., 1996). Transgenic studies showed that P2X1 receptors on platelets do not contribute to normal bleeding but play a significant role in thromboembolism models (P2X1 reduction was protective (Hechler et al., 2003) and over-expression of P2X1 receptors increased mortality (Oury et al., 2003)). Regulation of P2X1 receptor activity may therefore play an important role in a variety of physiological processes.

P2X1 receptors are co-expressed with G-protein coupled receptors in many tissues. For example, with ADP-sensitive P2Y_1_ G-protein coupled receptors on platelets (Gachet, 2006) and with ATP/UTP-sensitive P2Y_2_ G-protein coupled receptors on arterial smooth muscle (Ralevic and Burnstock, 1998). Activation of Gq coupled G-protein coupled receptors potentiated P2X1 receptor responses in arteries (Ase et al., 2005; La and Rand, 1993), as well as recombinant systems (Ase et al., 2005; Vial et al., 2004b), and provides a mechanism for fine tuning of responses. These effects are mimicked by the phorbol ester phorbol-12-myristate-13-acetate (PMA) and are dependent on activation of staurosporine sensitive protein kinases (Vial et al., 2004b; Wen and Evans, 2009). However, the lack of effect of mutation of the conserved protein kinase C site in the intracellular amino terminus of the P2X1 receptor on PMA or G-protein coupled receptor regulation, coupled with the lack of changes in the phosphorylation status of the P2X1 receptor following treatment suggests that the regulation occurs through effects on an interacting regulatory protein (Vial et al., 2004b). We have shown that residues in the intracellular amino terminus close to the first transmembrane segment (TM1) play an important role in P2X1 receptor regulation by PMA and Gq coupled receptors (Wen and Evans, 2009). Our recent studies have shown that PMA treatment also leads to an increase in P2X1 receptor mobility (Lalo et al., 2010). The carboxy terminus of P2X receptor contains a conserved trafficking motif YXXXK (Chaumont et al., 2004) and this raises the possibility that the carboxy terminus may also play a role in regulation of the receptor. In this study we have used over-expression of the intracellular C terminus and site directed mutagenesis based approach to demonstrate for the first time that the C terminal contributes directly to PMA and Gq coupled G-protein coupled receptor regulation of P2X1 receptors.

## 2 Materials and methods

### 2.1 Intracellular C terminus fragment construction

The carboxy terminal sequence (Leu^353^–Stop^400^) of the human P2X1 receptor was amplified from a pcDNA 3.0 vector containing the human P2X1 receptor cDNA by PCR (Techne Genius thermocycler, BioTAQ ™ DNA polymerase, Bioline, U.K.). Start and stop codons at the ends, as well as restriction sites EcoRI and HindIII, were introduced using primers. The sequence was ligated into the plasmid pcDNA3.0 using these two restriction sites at 14 °C overnight (T4 DNA ligase, New England Biolabs® Inc.).

### 2.2 Site-directed mutagenesis

Point mutations were introduced into the human P2X1 plasmid or the C terminal fragment construct using the QuikChange™ mutagenesis kit (Stratagene, Amsterdam, Netherlands) according to the manufacturer's instructions as described previously (Ennion et al., 2000) and confirmed by DNA sequencing (Automated ABI Sequencing Service, Leicester University, Leicester, U.K.).

### 2.3 Expression in *Xenopus laevis* oocytes

The human mGlu_1α_ receptor was a gift from Professor S. R. Nahorski (University of Leicester, Leicester, U.K.). pcDNA3.1 vectors (Invitrogen, Paisley, U.K.) containing either P2X1 mutant or wild type P2X1, mGlu_1α_ receptors or the C-termini mini-gene were linearized. Sense-strand cRNAs were generated from these linearized plasmids with the T7 mMessage mMachine™ kit (Ambion (Europe), Huntingdon, Cambs., U.K.). *Xenopus laevis* oocytes*,* stage V, were prepared by enzymatic treatment followed by manual defoliculation (Ennion et al., 2000). 50 nl of mRNA (1 μg/μl) was injected into *Xenopus* oocytes with an Inject + Matic microinjector (J. Alejandro Gaby, Geneva, Switzerland). For co-injections with the mini-gene the RNA was mixed to give 5 ng wild type P2X1 + 10 ng mGlu_1α_ receptor + 35 ng C-termini mini-gene (or appropriate volume of water was added in the absence of mini-gene) and injected in a 50 nl volume. Cells were maintained at 18 °C in ND96 buffer (concentrations in mM; 96 NaCl, 2 KCl, 1.8 CaCl_2,_ 1 MgCl_2_, 5 sodium pyruvate and 5 HEPES, pH 7.5) with 50 μg/ml gentamicin and were used for recording after 2–6 days.

### 2.4 Electrophysiological recordings

Two-electrode voltage clamp was used to record ATP (Mg salt; Sigma, Poole, UK) evoked currents as described previously (Ennion et al., 2000). ATP was applied with a fast-flow U-tube perfusion system and applications were separated by 5 min to allow recovery from desensitization. For *oocytes* pre-treated with PMA, 100 nM PMA was made in ND96 solution and the *oocytes* were pre-incubated in the PMA solution for 10 minutes at room temperature immediately before recording. Comparisons were made between groups of control untreated *oocytes* and those exposed to PMA. When looking at the potentiation of the P2X1 receptor, glutamate (100 μM) was bath-perfused for 5 min between ATP applications as described previously (Vial et al., 2004a,b).

### 2.5 Western blotting

The total and surface expression levels of P2X1 receptors were estimated by Western blotting as described previously (Ennion et al., 2000).

### 2.6 Data analysis

Data are shown as mean ± S.E.M. Significant differences between the means compared to wild type were calculated by one-way ANOVA, followed by Dunnett's test for comparisons of individual mutants against control using the GraphPad Prism 5 for Windows (GraphPad Software, San Diego, CA). Student's *t* test were also used where appropriate and considered to be significant when P < 0.05. *n* corresponds to the number of *oocytes* tested for electrophysiological data.

## 3 Results

### 3.1 The C terminus of the P2X1 receptor is involved in PMA and G-protein coupled receptor regulation

**Fig. 1:**
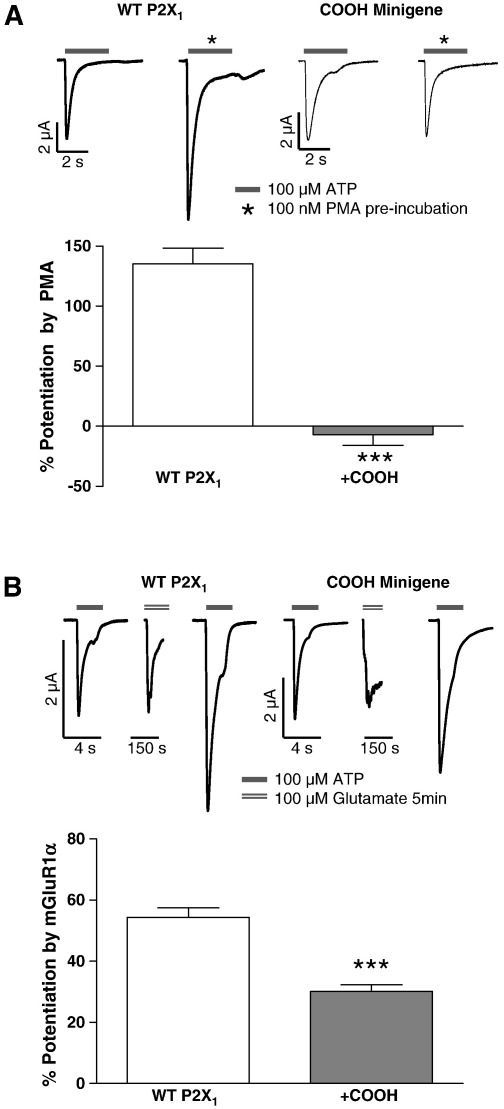
PMA and mGlu_1α_ receptor evoked potentiation of P2X1 receptors is blocked by over-expression of the intracellular C terminus. The carboxy terminus of the P2X1 receptor was co-expressed with wild type P2X1 and mGlu_1α_ receptors in *Xenopus oocytes*. (A) Upper left panels show representative currents evoked by a maximal concentration of ATP (100 μM, indicated by bar) at control *oocytes* (wild type P2X1) and those following 10 min incubation with PMA (100 nM). Right upper panels show the effects of over-expression of the interacellular carboxy terminus on the effects of PMA. The bar chart shows summary data, n = 5–15. (B) Upper panels show sample traces for a given oocyte co-expressing P2X1 and mGlu_1α_ receptors (left) or P2X1 receptors, mGlu_1α_ receptors and the P2X1 receptor carboxy terminal fragment (right traces). Responses to a maximal concentration of ATP (100 μM, indicated by bar) are shown before and after the application of glutamate (100 μM). Glutamate evoked an inward calcium activated chloride current and potentiated subsequent ATP evoked responses. This potentiation was reduced by co-expression of the P2X1 receptor C terminal mini-gene. The bar chart shows a summary of the data, n = 5–7. *** P < 0.001.

We have previously shown that staurosporine sensitive protein kinases are involved in the regulation of P2X1 receptors by PMA and stimulation of mGlu_1α_ receptors (Vial et al., 2004b; Wen and Evans, 2009). To determine whether the intracellular C terminus plays a role in this regulation of the P2X1 receptor we tested the effects of over-expression of the intracellular C terminus. If the C terminus is involved in regulation over-expression of this region would be expected to interfere with the modulation of the full length wild type receptor. The wild type P2X1 receptor was co-expressed with mGlu_1α_ receptors and the P2X1 receptor mini-gene at a ratio of 1:2:7. Over-expression of the C terminus had no effect on P2X1 receptor currents (peak current amplitudes to 100 μM ATP of − 6983 ± 340 nA, *n =* 5 for control and − 6959 ± 626 nA, *n =* 7 for carboxyl terminus over-expression), or the amplitude of glutamate evoked chloride currents (to 100 μM glutamate of − 5442 ± 777 nA, *n =* 5 for control, and − 5511 ± 1178 nA, *n =* 7 for carboxy terminus over-expression) demonstrating that over-expression of the C terminus does not affect P2X1 or mGlu_1α_ receptor expression, or activation. Treatment with either PMA (100 nM) or glutamate (100 μM, for 5 min before the application of ATP) potentiated P2X1 receptor currents as reported previously (Vial et al., 2004b; Wen and Evans, 2009). However over-expression of the C terminus reduced potentiation by PMA (from 135.3 ± 13.0%, *n =* 5 to −6.89 ± 9.08%, *n =* 15, ***P < 0.001) and mGlu_1α_ receptor stimulation (from 54.4 ± 3.0%, *n =* 5 to 30.1 ± 2.2%, *n =* 7, ***P < 0.001) (Fig. 1). These results suggest that the C terminus is involved in kinase regulation of the P2X1 receptor.

### 3.2 Effects of cysteine mutations in the C terminus of P2X1 receptor properties

**Fig. 2:**
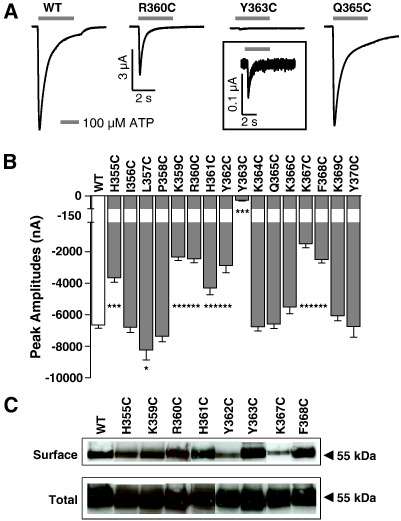
Properties of individual cysteine point mutations of the C terminus. (A) ATP (100 μM, application period indicated by bar) evoked rapidly desensitising currents at wild type P2X1 receptors. Cysteine mutation had no effect at the Q365C mutant but responses were reduced in amplitude by ~ 50% for R360C and by > 98% for Y363C. (B) Peak current amplitudes of ATP (100 μM) evoked currents from wild type (WT) and cysteine mutant P2X1 receptors, * P < 0.05, *** p < 0.001. (n = 4–18). (C) Total and surface expression levels of wild type and mutant P2X1 receptors with reduced peak current amplitudes are shown.

The effects of PMA on P2X1 receptor mobility (Lalo et al., 2010) suggest that the C terminal trafficking motif may be involved in regulation. We therefore generated a series of 16 cysteine point mutants (H355C to Y370C) that encompass the region between the end of the second transmembrane segment (TM2) and after the YXXXK trafficking motif. A transient inward current was evoked during the continued presence of ATP (100 μM, a maximal concentration) at wild type P2X1 receptors (−6442 ± 211 nA, *n =* 15). Peak current amplitudes were unaffected for 7 of the mutants, slightly potentiated by ~ 20% for L357C, reduced by ~ 30−80% for 7 mutants, and reduced by > 98% for Y363C (Fig. 2A,B). The decrease in peak current is unlikely to result from a major reduction in ATP sensitivity as increasing the concentration of ATP had no effect on peak current amplitude. To investigate whether the reduced current of the mutants resulted from decreased surface expression a sulfo-NHS-LC biotinylation assay (Ennion et al., 2000) was used to label the P2X1 receptors expressed on the surface of oocytes. Total and surface expression levels were visualized by Western blotting. The bands for the P2X1 receptors were revealed as monomers of ~ 55 kDa. Surface and total P2X1 receptor protein levels were comparable to wild type receptors for 6 of the 8 mutants with reduced peak currents (Fig. 2C). For Y363C the peak current was reduced by > 98% but there was no effect on the surface expression of the receptor indicating that this residue is likely to play an important role in the activation/gating of the receptor. Reduction in surface expression (> 50%) was observed at Y362C and K367C mutants suggesting these are important for protein trafficking. These results highlight the contribution of the C terminus to the expression and activation of P2X1 receptors.

### 3.3 Contribution of C terminal residues to PMA and G-protein coupled receptor regulation

**Fig. 3:**
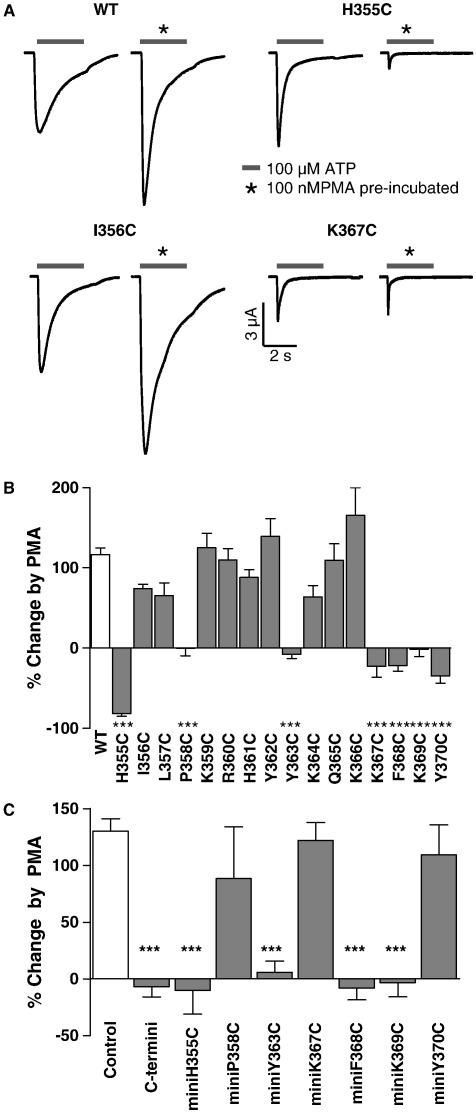
Effects of cysteine mutations in the carboxy terminus of P2X1 receptors on PMA potentiation. (A) Representative traces of ATP evoked currents (100 μM, application indicated by bar) from oocytes under control conditions and following treatment with PMA (100 nM) for wild type (WT) and mutants H355C, I356C and K367C. (B) Summary of the percentage change in peak current amplitude to ATP following PMA treatment for P2X1 receptor cysteine mutants (n = 4–18). (C) Effects of cysteine mutations that reduced PMA potentiation when introduced into the P2X1 receptor when introduced into the C terminal fragment. Cysteine mutations P358C, K367C and Y370C abolished the inhibitory effect of the C terminal fragment on PMA regulation. (n = 5–15).

Treatment with PMA (100 nM) evoked a similar potentiation to that recorded for P2X1 receptors (116.5 ± 14.5%, *n =* 11) for the mutants I356C, L357C, K359C, R360C, H361C, Y362C, K364C, Q365C and K366C indicating that these residues do not play an essential role in PMA regulation. In contrast, potentiation was abolished for the mutants P358C, Y363C, K367C, F368C, K369C and Y370C. P2X1 receptor currents were inhibited by ~ 80% for the mutant H355C, (Fig. 3, ***P < 0.001). Similar reductions in mGlu_1α_ receptor evoked potentiation were also seen for these mutants (data not shown). These results show that individual residues close to the second transmembrane segment, and around the YXXXK protein trafficking motif, are involved in P2X1 receptor regulation.

Over-expression of the C terminus abolished PMA regulation and this could result from sequestering of a regulatory interacting protein. This is consistent with our previous studies that suggested that protein kinase mediated regulation of the receptor is likely to be through phosphorylation of an interacting protein (Vial et al., 2004b). This could involve two steps, firstly the binding of the interacting protein to the P2X1 receptor, and secondly the transduction of the effects of the phosphorylated protein to the increase in P2X1 receptor responsiveness. It is possible that different amino acids could be involved in the binding of the interacting protein and the subsequent transduction event. If this is the case mutation of residues involved in binding the interacting protein should abolish the sequestering effect of over-expression of the C terminal fragment but residues involved in transduction would have no effect on the effectiveness of the fragment. Therefore the cysteine mutants that inhibited the PMA effect were introduced individually into the C terminal fragment and tested (Fig. 3C). Over-expression of C terminal fragments incorporating the mutants H355C, Y363C, F368C and K369C still inhibited the PMA potentiation. However C terminal fragments with either the P358C, K367C or Y370C mutation had no effect on PMA potentiation. These results suggest that residues P358, K367 and Y370 are involved in direct interaction with a regulatory factor and residues H355, Y363, F368 and K369 are involved in transduction of the regulatory event.

## 4 Discussion

The present study shows that the C terminus plays an important role in the regulation of P2X1 receptor expression, gating, and regulation by PMA and Gq G-protein coupled receptors. Point mutations in the region close to TM2 incorporating a conserved trafficking motif can lead to a reduction in P2X1 receptor responsiveness. For the H355C, K359C, R360C, H361C, Y363C and F386C mutants surface expression of these mutants was unaffected and it is likely that the reduction in responsiveness resulted from a reluctance of the ATP bound receptor to open i.e. an effect on channel gating. This is consistent with previous studies showing that the C terminus contributes to shaping the time-course of P2X receptor currents (Brandle et al., 1997; Fountain and North, 2006; Smith et al., 1999). For the mutants Y362C and K367C the ~ 50% reduction in ATP evoked currents was mirrored by an equivalent reduction in their surface expression. At first glance this appears consistent with the role tyrosine and lysine residues in the YXXXK conserved trafficking motif identified in P2X2-6 receptors (Chaumont et al., 2004). However if this were the case it should be Y363C that had reduced surface expression; this mutant showed a > 98% reduction in evoked currents but no change in surface expression. These results suggest that at the P2X1 receptor there is some flexibility in the trafficking motif and that it is a YXXXXK and not a YXXXK motif that regulates their surface expression.

Over-expression of protein fragments can interfere with receptor regulation. The reduction of PMA and G-protein coupled receptor potentiation of the P2X1 receptor following over expression of the C terminal domain identified the importance of this region in channel regulation. We and others have previously shown that P2X1 receptor potentiation by PMA and G-protein coupled receptors was mediated by a novel serine/threonine protein kinase C isoform (Ase et al., 2005; Wen and Evans, 2009). There are no consensus sites for protein kinase C phosphorylation in the carboxy terminus. However the cysteine mutations H355, P358, Y363C and K367-Y370 abolished the potentiation by PMA and this suggests the receptor is regulated through an indirect mechanism. This further supports previous studies demonstrating that removal of the consensus sequence for PKC phosphorylation in the amino terminus has no effect on potentiation, and that a change in P2X1 receptor phosphorylation could not be detected following PMA treatment (Vial et al., 2004b), leading to the conclusion that protein kinase C regulates the P2X1 receptor through phosphorylation of an interacting regulatory protein (Vial et al., 2004a; Wen and Evans, 2009). By combining over-expression of the intracellular C terminal fragment and mutagenesis approaches we were able to characterise these cysteine mutations into those that had no effect on (H355C, Y363C, F368C and K369C), or abolished the inhibitory effects of the C terminal fragment (P358C, K367C and Y370C). These results highlight the importance of residues P358, K367 and Y370 in the sequestering effects of the C terminal fragment and suggest that they may interact directly with the regulatory protein. In contrast the abolition of PMA and G-protein coupled receptor regulation by mutations H355C, Y363C, F368C and K369C when they were introduced into the full length P2X1 receptor and but not the C terminal fragment suggests that these residues are not involved in direct interaction with the regulatory factor but are essential for transduction of the change evoked by the regulatory factor.

**Fig. 4:**
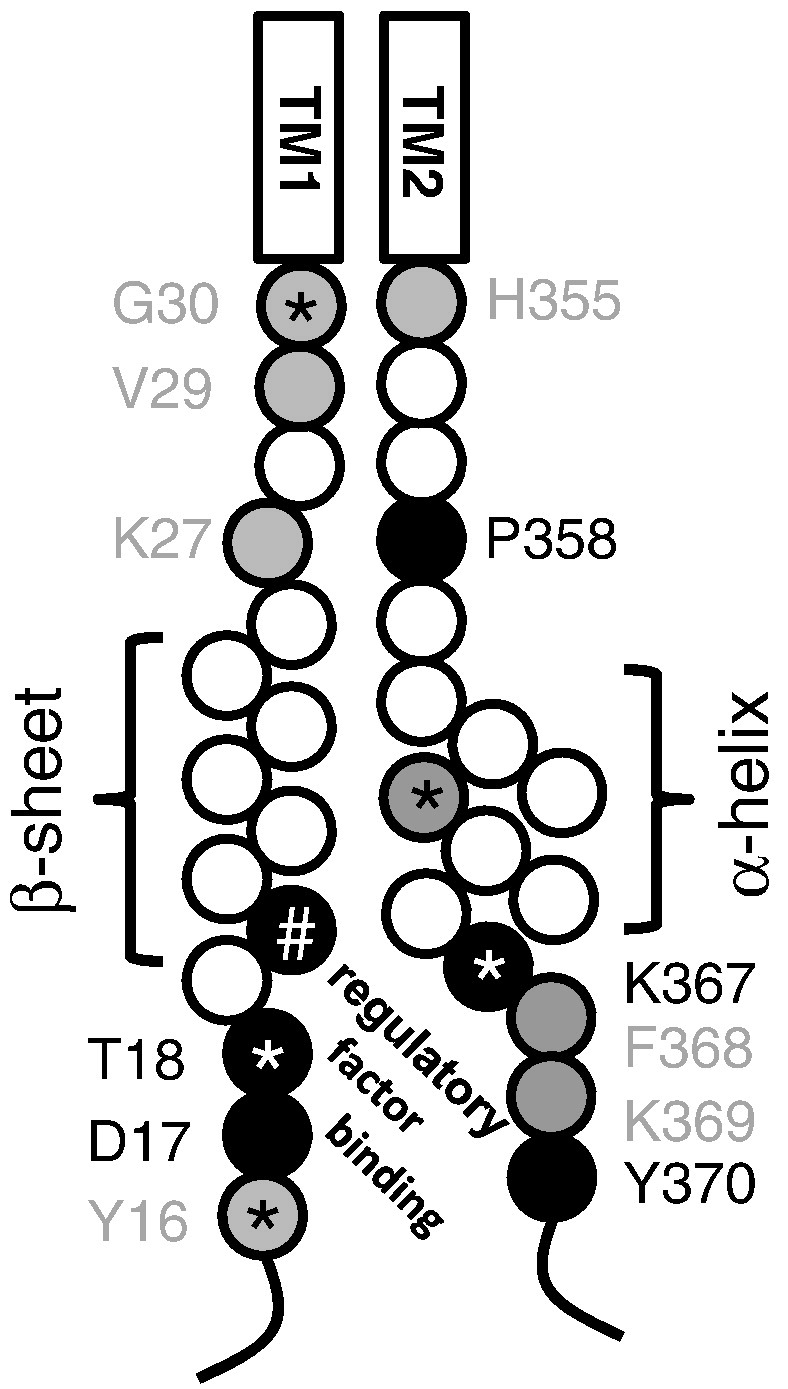
Model of the amino and carboxy termini of the P2X1 receptor showing residues involved in PMA regulation. The effects of cysteine mutants of residues Y16-G30 (from (Wen and Evans, 2009)) and H355-Y370 on PMA regulation are shown. Residues in back abolished PMA regulation when introduced into the P2X1 receptor and removed the inhibitory effect of the corresponding mini-gene (involved in sequestering effect of mini-gene and predicted interaction with regulatory protein). Residues in grey are those that when mutated in the P2X1 receptor abolished PMA regulation but did not reduce the inhibitory effect when introduced into the C terminal fragment (transduction effect). The transmembrane segments are shown as boxes (TM1 and TM2). Secondary structural predictions of a beta sheet in the amino terminus and an alpha helix in the carboxy terminus are shown, * indicates residues that are conserved throughout the P2X receptor family and # indicates where there is a conserved positive charge.

We have previously shown that the amino terminus of the P2X1 receptor also contributes to PMA and G-protein coupled receptor mediated potentiation of the receptor (Wen and Evans, 2009). This work, like the current study, also identified residues that were proposed to be involved in binding of a regulatory factor(s) and those involved in transduction of the regulatory event. This raises the possibility that the intracellular amino and carboxy termini may interact to regulate the receptor. Structural information on this interaction is not currently available as the intracellular domains were truncated to facilitate the crystalization of the zebra-fish P2X4 receptor (Kawate et al., 2009). However a homology model of the P2X1 receptor based on the zebra-fish P2X4 receptor structure predicts that residues G30 and H355 are ~ 14 angstrom apart. Bio-informatic algorithms predict that part of the amino terminus before TM1 forms a beta sheet and a region of the carboxy terminus after TM2 may form as an alpha helix ((McGuffin et al., 2000). Based on this information we can map the mutagenesis results of this, and our previous study on PMA regulation (Wen and Evans, 2009) to produce a model of the intracellular domains (Fig. 4). These results demonstrate that residues involved in PMA regulation are clustered. It is interesting that those amino acids that when mutated to cysteine and abolish PMA regulation when introduced to the full length P2X receptor, and negate the action of the amino or carboxy termini mini-genes, are located equidistant (11–14 and 14–17 residues) from the TMs. It is tempting to speculate that these residues, involved in the sequestering action of the mini-genes, may interact to form a pocket/environment for the binding of an interacting protein and regulation by PMA. Interestingly this brings together the two intracellular regions (TXR/K in the amino terminus and YXXXK in the carboxy terminus) that are conserved throughout the P2X receptor family. Indeed, where tested, other P2X receptor subtypes have also been shown to be regulated by PMA/Gq coupled G-protein coupled receptors (Boue-Grabot et al., 2000; Paukert et al., 2001). This suggests that this region may form a conserved pocket for P2X receptor regulation. The region within 1–4 amino acids of the TM domains also comprises a cluster of residues that regulate PMA action through an effect when introduced in the P2X1 receptor but not the C terminal fragment; indicative of an effect on transduction of the regulatory event. There is one exception; P358C. However proline is a unique amino acid that can introduce kinks in proteins and mutation to cysteine may have had an adverse effect on the folding of the C terminal fragment that could explain the reduction of effect. Overall these results suggest that the intracellular amino and carboxy termini interact and form a binding pocket for a regulatory factor that can potentiate P2X1 receptor currents. This provides a mechanism for the fine tuning of the responsiveness of the P2X1 receptor in native systems by G-protein coupled receptors, for example in arteries and platelets.
